# Report of a Case of Imperforate Rectum, Sucessfully Operated upon

**Published:** 1855-10

**Authors:** R. A. F. Penrose


### *Report of a Case of Imperforate Rectum, sucessfully operated upon.* By R. A. F. Penrose, M.D.

The child who possessed the peculiar conformation alluded to in the heading of our report, was born on the 10th of July, 1855. Upon examination, after birth, it presented nothing unusual, being a boy, and apparently well formed in every respect. Visiting the mother next day, and finding its bowels had not been opened, some of the ordinary remedies were directed with the object of procuring a passage, and these failing, a dose of castor oil was given, but with like results.

On the following day, the child's bowels still being unmoved, I resolved to administer to it myself an injection into the rectum; the end of the syringe passed the anus without difficulty, but, to my surprise, the whole of the injection returned as fast as forced up. A careful examination was now made, which led to the discovery of the obstruction.

Externally, the anus was found perfectly formed; a bougie introduced into it passed the sphincter without difficulty, but was arrested after having penetrated about an inch and a quarter, the rectum terminating here in a blind pouch.

The propriety of an operation being undoubted, it was determined to perform it at once, the condition of the child demanding, if possible, immediate relief. It had become, by this time, very restless and uneasy, strained frequently and violently, as if trying to evacuate the contents of its bowels; its belly was, also, much swollen and discolored; until this day, it had taken the breast well, but now refused it, exhibiting every mark of suffering, and no little nervous irritation.

The operation was performed in the following manner:

The child being held in the lap of an assistant, a finger was introduced into the rectum until arrested by the termination of the gut; an exploring needle in a canula was now slid along the finger, and forced into the septum, in the direction in which the gut should run. This structure proved tough and resisting, yielding and slipping to one side, rather than allowing the needle to pass. After several ineffectual attempts, the needle at last passed through the obstruction, on withdrawing it, and then the canula, a very small drop of meconium was found in the tube of the latter. Through the passage thus made with the exploring needle, a grooved director was now passed; along the groove of this director another was slid, and then rotated so as to bring its groove opposite the groove of the first director, in other words, making a tube through which the meconium might flow; at once this began slowly to be discharged. To facilitate its passage, and, at the same time, to enlarge the opening, a small piece of wood was now placed between the two directors near the anus, and then by pressing the handles together, the opposite ends were separated considerably, and the opening much dilated. The meconium began at once to pass profusely and without difficulty, to the immediate relief of the child.

Keeping the directors in the opening, a very large gum elastic bougie was now passed up between them, and finally this was withdrawn, and the little finger introduced through the opening made, tearing and dilating it still more, until it was almost of the same diameter as the portion of intestine above and below it.

The septum through which the opening was made, seemed, as nearly as could be determined by the finger, about a quarter of an inch in thickness, of a firm, fibrous or cartilaginous character, the gut immediately above and below it being of natural size.

During all these manipulations, which must have occupied half an hour at least, the child seemed to suffer very little, being apparently almost entirely occupied with its efforts to evacuate its accumulated meconium through the passage made; the principal sign of uneasiness being hiccough, as the strictured part was being torn and dilated.

A copious discharge from the bowels having now taken place, a well greased plug of lint was passed up to the strictured part, the lower portion being allowed to protrude through the anus, and the sixth of a drop of laudanum was given to the child, with directions to repeat it as often as might be necessary through the night.

A few extracts from the notes on the case will complete its history:

July 14th.—The child has had quite a comfortable night, having taken altogether about three quarters of a drop of laudanum. Bowels open several times without much straining; belly not nearly so much distended; child disposed to take the breast. Another greased plug introduced; laudanum continued; at night a fresh plug.

July 15th.—Child still does well; belly almost natural size; sleeps most of the time; bowels opened frequently. As the plug seemed to give rise to a good deal of irritation of the rectum, it was now dispensed with, and the introduction of a bougie twice a day adopted as a substitute.

From this time, the child continued to do well; its treatment being the introduction of a large bougie, twice daily, except when it seemed to irritate too much; the use of opium, taking from one, to one and a half drops of laudanum in twenty-four hours, for almost two weeks, after which it was gradually omitted.

The gum bougie was used daily for two weeks, after that, less and less frequently, until at the present time, when it is used about every eight or ten days. The child now, ten weeks after the operation, is fat and healthy, its bowels being open naturally and without difficulty.

The advantages of the operation above detailed, for similar cases, consists in its great safety; the only puncture made with the exploring needle, being so small, that if it even should penetrate into the cavity of the abdomen, the injury might prove comparatively harmless; whilst as soon as it has passed into the extremity of the gut beyond, a way is opened for the introduction of directors, and the subsequent enlargement accomplished by dilating and tearing, and not by cutting.

